# Validation of a novel wearable, wireless technology to estimate oxygen levels and lactate threshold power in the exercising muscle

**DOI:** 10.14814/phy2.13664

**Published:** 2018-04-02

**Authors:** Parisa Farzam, Zack Starkweather, Maria A. Franceschini

**Affiliations:** ^1^ Athinoula A. Martinos Center for Biomedical Imaging Massachusetts General Hospital Harvard Medical School Boston Massachusetts

**Keywords:** Athletic exercise training, blood lactate, muscle oxygen saturation, near‐infrared spectroscopy (NIRS)

## Abstract

There is a growing interest in monitoring muscle oxygen saturation (SmO_2_), which is a localized measure of muscle oxidative metabolism and can be acquired continuously and noninvasively using near‐infrared spectroscopy (NIRS) methods. Most NIRS systems are cumbersome, expensive, fiber coupled devices, with use limited to lab settings. A novel, low cost, wireless, wearable has been developed for use in athletic training. In this study, we evaluate the advantages and limitations of this new simple continuous‐wave (CW) NIRS device with respect to a benchtop, frequency‐domain near‐infrared spectroscopy (FDNIRS) system. Oxygen saturation and hemoglobin/myoglobin concentration in the exercising muscles of 17 athletic individuals were measured simultaneously with the two systems, while subjects performed an incremental test on a stationary cycle ergometer. In addition, blood lactate concentration was measured at the end of each increment with a lactate analyzer. During exercise, the correlation coefficients of the SmO_2_ and hemoglobin/myoglobin concentrations between the two systems were over 0.70. We also found both systems were insensitive to the presence of thin layers of varying absorption, mimicking different skin colors. Neither system was able to predict the athletes’ lactate threshold power accurately by simply using SmO_2_ thresholds. Instead, the proprietary software of the wearable device was able to predict the athletes’ lactate threshold power within half of one power increment of the cycling test. These results indicate this novel wearable device may provide a physiological indicator of athlete's exertion.

## Introduction

Quantifying how muscles respond to physical exercise is of great interest to athletes for improving performance and mitigating the risk of injury. Traditionally, athletes rely on measurements such as heart rate, blood lactate concentration, or maximum oxygen uptake (*V*O_2max_). These parameters are used to determine the intensity levels at which athletes should be exerting themselves to maximize athletic performance (Seiler and Kjerland 2006; Bentley et al. 2007; Esteve‐Lanao et al. 2007; Goodwin et al. 2007). Although heart rate, blood lactate concentration, and *V*O_2max_ can help guide an athlete's training regimen, these measurements are indicative of systemic changes occurring in the body, with no specific information about the working muscles.

Interest in examining muscle oxygen saturation (SmO_2_) has been growing due to its ability to provide a localized measurement continuously and noninvasively using near‐infrared spectroscopy (NIRS) (Chance et al. 1992; Belardinelli et al. 1995; Hamaoka et al. 1996, 2011; Bhambhani et al. 1997; Grassi et al. 1999, 2003; Wang et al. 2006; Soller et al. 2008; Bailey et al. 2009; Ihsan et al. 2013; Racinais et al. 2014; Boone et al. 2016; van der Zwaard et al. 2016; Baker et al. 2017; Hammer et al. 2018; Perrey and Ferrari 2018). NIRS techniques work by delivering light (in the 650–900 nm wavelength range) into the tissue and measuring the diffused light to estimate the absorption and scattering properties of the measured tissue volume (Yodh and Chance 1995). The concentrations of oxyhemoglobin (HbO) and deoxyhemoglobin (HbR) in the tissue can be estimated from the measured absorption spectrum. Muscle oxygen saturation, also referred as muscle oxygenation (SmO_2_), is then calculated by taking the ratio of HbO to total hemoglobin concentration (HbT). Previous studies have investigated the HbO, HbR, HbT, or SmO_2_ trends during exercise to determine if these NIRS parameters provide useful information to guide athletic training (Belardinelli et al. 1995; Bhambhani et al. 1997; Grassi et al. 1999, 2003; Wang et al. 2006; Bailey et al. 2009; Hamaoka et al. 2011; Racinais et al. 2014). Correlations have been observed between the threshold power found from *V*O_2max_ or lactate data and NIRS methods (Belardinelli et al. 1995; Grassi et al. 1999; Wang et al. 2006; van der Zwaard et al. 2016). These NIRS studies developed techniques to find an athlete's threshold power by analyzing the trends in HbO, HbR, HbT, or SmO_2_ after an incremental power test (Belardinelli et al. 1995; Grassi et al. 1999; Wang et al. 2006; van der Zwaard et al. 2016). Other work has been performed to understand muscle adaptations throughout an athlete's training by examining the HbO, HbR, HbT, or SmO_2_ kinetics during specific exercise protocols (Bailey et al. 2009; Ihsan et al. 2013). These studies support the incorporation of NIRS technology into athletic training but are limited to the laboratory setting due to the required optical fibers, probes, and larger instruments to obtain the measurements. The noninvasive monitoring of muscle oxygenation could significantly benefit from small, wearable, wireless, and accurate devices that can deliver real‐time feedback to athletes.

Currently, there are a small number of wearable, fiber‐less, NIRS devices used in the athletic market, such as the Portamon (Artinis Medical System, Einsteinweg, The Netherlands), Moxy Monitor (Fortiori Design, LLC, Hutchinson, MN, USA), and BSX Insight (BSX Athletics, Austin, TX, USA). A review from Perrey and Ferrari (2018) goes into detail about the different studies that have examined the use of these NIRS devices in athletic training and SmO_2_ monitoring. The Portamon and Moxy devices can be manually strapped on to any muscle group and have been used during a variety of activities, including cycling, running, and strength training (Perrey and Ferrari 2018). The BSX Insight is designed to be worn on the gastrocnemius muscle within a custom‐made compression sleeve and differentiates itself by providing athletes with their lactate threshold when following a given protocol (Borges and Driller 2016; Driller et al. 2016). These studies on wearable NIRS devices in the athletic community show value in obtaining optical measurements during sports, however the validity of measurements from wearables would be enhanced by a direct comparison with a bench‐top, fiber‐based, FDNIRS system.

In this study, we compared the SmO_2_ of the quadriceps muscle group recorded by a wearable, low‐cost, continuous‐wave (CWNIRS) consumer device (Humon Beta, Dynometrics, Inc.) against a benchtop fiber‐based frequency‐domain near‐infrared spectroscopy (FDNIRS) system (MetaOx, ISS, Champaign, IL). The FDNIRS system is considered the most robust and reliable reference commercially available for comparison of oximeters (Kleiser et al. 2016). Our goal was to examine the accuracy of this wearable device and understand the limitations that arise when using CWNIRS for muscle oxygenation measurements. We also investigated the real‐time feedback from the Humon Beta device and reported the differences between the optically derived threshold and blood lactate threshold during an incremental cycling test.

## Methods

### Study population and measurement protocol

**Figure 1:**
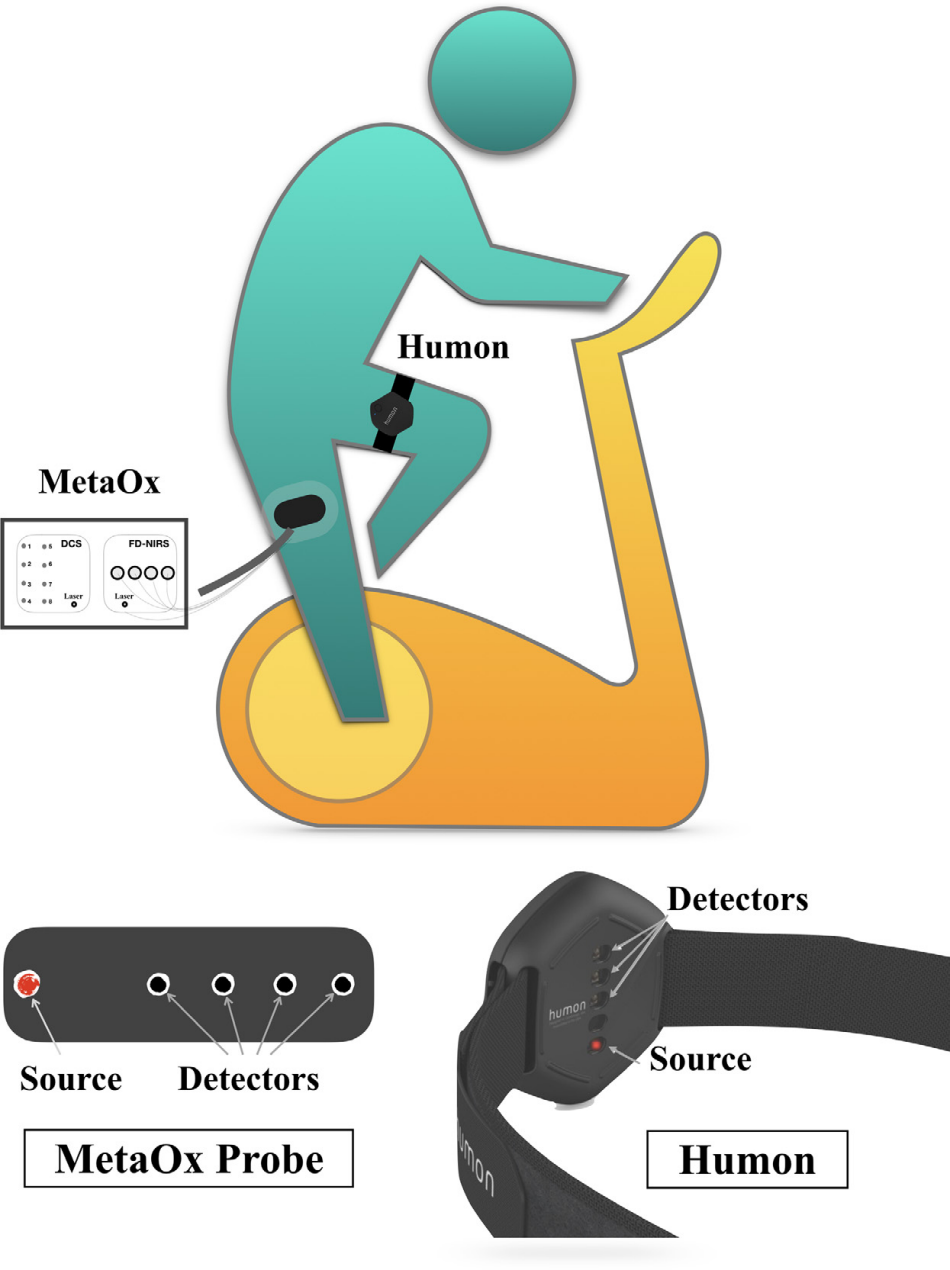
The schematic of the measurement on an upright stationary ergometer. The top drawing displays the location of Humon wearable on the left leg and MetaOx probe on the right leg. The distribution of the sources and detectors are presented for both the MetaOx probe (source–detector separations: 1.5, 2.0, 2.5, and 3.0 cm) and Humon Beta wearable (source–detector separations: 1.2, 1.8, and 2.4 cm) in the bottom images.

Fifteen male and three female athletic subjects performed an incremental step test on the cycle ergometer. For logistical reasons, the test was carried out with the MetaOx probe on the right leg rectus femoris and the Humon Beta on the left leg rectus femoris, as illustrated in Figure 1. The rectus femoris was chosen as the muscle to monitor in order to minimize fiber movement since this was the area of the leg where the fibers could remain most stable. Each session began with baseline measurements for approximately two minutes, where subjects were instructed to stay as still as possible. Following a well‐established incremental test protocol (Madden et al. 2013), the subjects began cycling at 30 W for 4 min, and the power was increased in 30 W increments every 4 min until voluntary exhaustion. Voluntary exhaustion was determined when the subject requested to stop or when he/she could no longer maintain the cycling cadence. In the last minute of each power interval, the blood lactate concentration was measured using a handheld lactate analyzer (Lactate Plus meter, Nova Biomedical, Waltham, MA) by averaging three repeated blood samples. The subjects were asked to keep a consistent cadence throughout the cycling protocol, which was typically between 80 and 100 rotations per minute (RPM). For each subject, the lactate threshold power was determined as the cycling power at which the athlete's blood lactate concentration reaches 4 mmol/L (Faude et al. 2009; Madden et al. 2013). One male subject was excluded from all data analyses because of a Humon Beta malfunction. On this subject, sweat entered into the sensor and corrupted the data, however, we verified that sweat did not enter on any other Humon Beta device and did not affect any other measurements. Thus, from now on, we just report the values of the 17 subjects. In seven of the subjects, two Humon Beta sensors were worn on the left rectus femoris muscle to examine the heterogeneity of the measurements within the same muscle. The devices were placed 2 cm apart from one another, where one was proximal and the other was distal along the muscle and the variability due to location placement was assessed. We verified the separation of the two devices was sufficient to prevent any light leakage between them.

Before beginning the cycling on an upright stationary cycle ergometer (Model E3, Kettler USA), basic information was collected for each subject. This information included age, height, weight, and gender. Body Mass Index (BMI) was calculated as (kg/m^2^). In addition, the subcutaneous adipose tissue thickness (SCATT) on the rectus femoris of the right quadriceps was measured for each subject using a skinfold caliper before the start of the cycling test. Information about the subjects can be found in Table 1. We also tested the vitals (heart rate, blood pressure, body temperature, arterial oxygen saturation) of all subjects to ensure that the subjects were healthy prior to the onset of exercise.

**Table 1:** Subjects demographic

	All	Males	Females
Number	17	14	3
Right‐footed	15	12	3
Age (years)	31 ± 6	30 ± 6	35 ± 5
Weight (kg)	71 ± 14	75 ± 11	51 ± 3
Height (cm)	176 ± 11	180 ± 7	156 ± 3
BMI (kg/m^2^)	23 ± 3	23 ± 3	21 ± 2
SCATT (mm)	5 ± 2	5 ± 2	7 ± 1

Details about the subjects' footedness, age, weight, height, BMI, and subcutaneous adipose tissue thickness (SCATT) are shown, including a gender breakdown for each category.

The study protocol was reviewed and approved by the Institutional Review Board (IRB) for Partners Healthcare, the Partners Human Research Committee (PHRC). The study method was designed and carried out in accordance with PHRC requirements and the regulations that govern human subjects research. All subjects interested in participating in the study went through a prescreening process to ensure they were eligible. These subjects all exercised more than 3 times per week, each session over 30 min and their exercise routine typically included biking, running, swimming, or rowing. We refer to this population as athletes throughout the text. They were all comfortable exercising at a high intensity for a long period of time. All eligible participants read and signed the approved informed consent form before starting the measurement session.

### Instrument setup

The Humon Beta device (Fig. 1) uses two light sources in the NIR window and three photodetectors to measure the intensity of the light that has propagated through the tissue. The sources and detectors are found behind individual polycarbonate windows, which come in contact with the skin of the user. The photodetectors are located at distances of 1.2, 1.8, and 2.4 cm from the light sources. The acquisition rate is set to 4 Hz. The Humon Beta is 6.0 × 5.7 × 1.4 cm in size and has a slight curvature in the plastic case to allow for easy contact with the skin on the quadriceps. The wearable is attached to the athlete's quadriceps, using a strap that hooks through the device and can be secured around the thigh with a hook‐and‐loop fastener. The Humon Beta communicates with a smartphone via Bluetooth, and a custom app displays the workout progress in real time.

MetaOx is a hybrid device that consists of FDNIRS to measure hemoglobin concentration and tissue oxygenation and diffuse correlation spectroscopy (DCS) to measure blood flow (Boas et al. 2016). The details of the system and data analysis are described by Carp et al. (2017). For this work, we only considered the FDNIRS data even though DCS data were also acquired. In summary, the FDNIRS components include 8 lasers in the visible and NIR spectral region (672, 726, 759, 813, 690, 706, 784, and 830 nm) modulated at 110 MHz and 4 photomultiplier tubes detectors (PMT) modulated at 110.005 MHz to achieve heterodyne detection at 5 kHz. The lasers are rapidly multiplexed in sequence and allow the fast measurement (10 Hz) over all wavelengths. The light is delivered to the tissue through a fiber optics bundle coupled to the probe. The diffused photons are collected by the probe's fiber optics bundles located at distances of 1.5, 2.0, 2.5, and 3.0 cm from the source (*ρ*), (see Fig. 1) and delivered to the four PMTs.

### Data analysis

The Humon Beta and MetaOx data were coregistered and acquired continuously for the whole duration of the protocol. For MetaOx data analysis, the detected light amplitude (AC) and phase shift at four separations and eight wavelengths are calibrated on a silicone tissue mimicking phantom with known optical properties to account for the different gains of the four detectors (Farzam et al. 2013). After calibration, the frequency‐domain solution of the photon diffusion equation in the semi‐infinite geometry is used to fit for the wavelength‐dependent optical properties of the tissue (Durduran et al. 2010). At each wavelength, a linear fit is performed on (ln(AC(*λ*)·*ρ* ^2^)) and phase over source‐detector distances. Absorption (*μ*_a_) and reduced scattering (*μ*_s_′) coefficients at each wavelength are calculated from the slopes of the linear fits (Fantini et al. 1995). The measured absorption coefficient is sum of the absorption of tissue chromophores (*μ*_a_ (*λ*) = ∑ *ε*_i_(*λ*)·c_i_), where the wavelength‐dependent extinction coefficient, *ε*_i_(*λ*), of the *i*th chromophore is obtained from the literature (Prahl), and c_i_ is the concentration of the *i*th chromophore. The primary muscle chromophores in the near‐infrared are water, oxy‐, deoxy‐hemoglobin, and myoglobin. The muscle tissue water percentage is assumed to be 75% (Franceschini et al. 1997). Since myoglobin cannot be distinguished from hemoglobin due to the spectral overlap (Quaresima et al. 2004), its contribution is combined into the calculated oxy‐ and deoxy‐hemoglobin concentrations. When we refer to hemoglobin throughout the text, we acknowledge that this is a combination of hemoglobin and myoglobin. The total hemoglobin concentration and oxygen saturation are calculated as HbT = HbO + HbR and SmO_2_ = HbO/HbT, respectively.

The Humon Beta is a CW device and only measures light intensity at three separations and two wavelengths. Hence, to estimate hemoglobin concentration and oxygenation, it needs to assume a fixed scattering coefficient. The algorithms used to recover hemodynamic parameters were not disclosed and are proprietary to Dynometrics Inc. The HbO, HbR, HbT, and SmO_2_ results obtained with the two devices were compared to determine the drawbacks of using a limited set of wavelengths and fixed scattering coefficients for a small, inexpensive wireless NIRS device which cannot include as many features as the benchtop frequency‐domain system.

In addition, to further estimate the error introduced by the simplifications in the wearable system, we compared full MetaOx results with estimates obtained, using a subset of the MetaOx intensity data, which best matches the Humon Beta separations and wavelengths. In this way, we can isolate the errors due to the reduced dataset from contamination arising from other parameters such as differences in sensor location and differences between left and right leg muscles.

To test the effect of superficial thin layers, such as different skin tones, on the data collected by the Humon Beta and MetaOx systems, neutral density (ND) filters were placed over a silicone tissue mimicking phantom with known optical properties and data were collected with the two systems. We evaluated how the presence of these superficial thin layers affect the recovered “effective SmO_2_”, both with the Humon Beta and the MetaOx. We call the calculated parameter “effective SmO_2_”, since the phantom is made of silicon and does not contain blood. For the MetaOx we also evaluated how the ND filter affected the measured intensity and the slope of both phase and intensity versus distance.

Moreover, in the 17 subjects we evaluate the effect of subcutaneous adipose tissue on the estimated HbO, HbR, HbT, and SmO_2_ by analyzing the MetaOx cycling data using only the shorter (1.5 and 2.0 cm) or the larger (2.5 and 3.0 cm) separations. We estimated the difference in the recovered HbO, HbR, HbT, and SmO_2_ at the two separations throughout the whole cycling test, and the correlation between SmO_2_ drop and measured SCATT.

Finally, we examined the relationship between the measured hemodynamic parameters and the blood lactate measures and tested the accuracy of the Humon Beta for predicting the lactate threshold power. The Humon Beta employs a proprietary algorithm to determine the lactate threshold in real time. The data are processed and displayed on a smartphone application, where in addition to SmO_2_ values, a corresponding exercise zone is shown. Four zones are used to classify the subjects exercise state: (1) the green zone represents a steady state, (2) the orange zone indicates the athlete is approaching their limit, (3) the red zone shows the athlete has hit/exceeded their limit, and (4) the blue zone means the athlete is in a recovery phase. For this analysis, we excluded the three female subjects since the power increments were too large, which resulted in the lactate threshold power and the end of the exercise to be too close in time.

For HbO, HbR, HbT, and SmO_2_ comparisons between the MetaOx and Humon Beta, we report the Pearson correlation coefficient and the relative root mean square error (RMSE). Nonparametric statistical tests were performed when comparing features in the SmO_2_ curves between the MetaOx and Humon Beta. All the data processing and statistical analyses in this paper are performed using MATLAB (MathWorks, USA), version R2017a, and Statistics and Machine Learning Toolbox, version 11.1.

## Results

### Optical and physiological properties of the leg muscle

**Figure 2:**
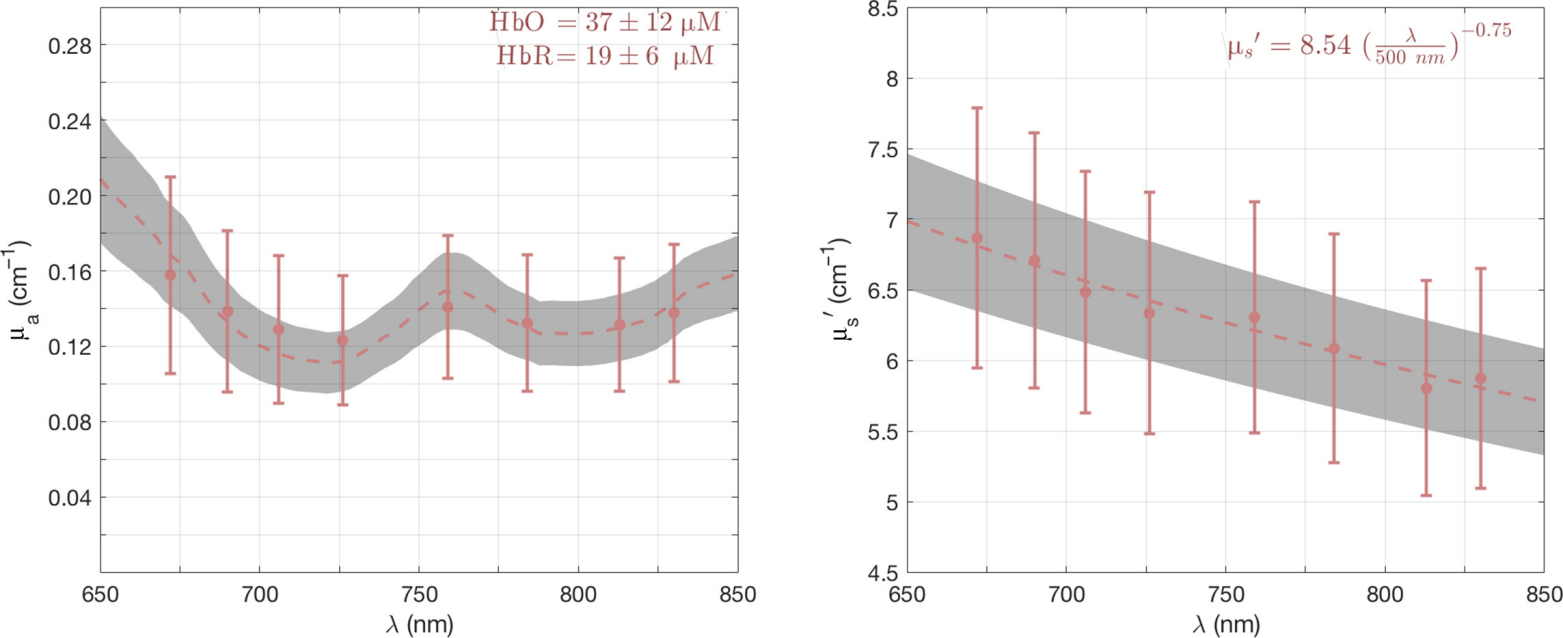
The measured absorption coefficient (*μ*_a_) and reduced scattering coefficient (*μ*_s_’) of rectus femoris muscle, and their fitted spectrum. The red error bars indicate the mean over all subjects and 95% confidence interval of the mean. The dashed red line is the fitted spectrum and the gray shaded area indicates 95% confidence interval of the fitted spectrum.

Figure 2 shows the average of optical properties, that is, the absorption coefficient (*μ*_*a*_) and reduced scattering coefficient (*μ*_s_′), measured by MetaOx across all the subjects over 2 min of baseline before starting to pedal. The error bars indicate the mean over all subjects and 95% confidence interval of the mean. The dashed red line is the fitted spectrum and the gray shaded area indicates the 95% confidence interval of the fitted spectrum. The resulting average oxy‐ and deoxy‐hemoglobin concentrations are 37 ± 12 (*μ*mol/L) and 19 ± 6 (*μ*mol/L), respectively. For the scattering, the average number density (a) is 8.54 ± 1.4 and the average effective particle size (b) is 0.75 ± 0.24, for the relationship derived from the Mie model (Jacques 2013).

The dashed red line is the mean of fitted spectrum and the gray shaded area indicates the 95% confidence interval of the mean.

### SmO_2_ trend during cycling incremental power test

**Figure 3:**
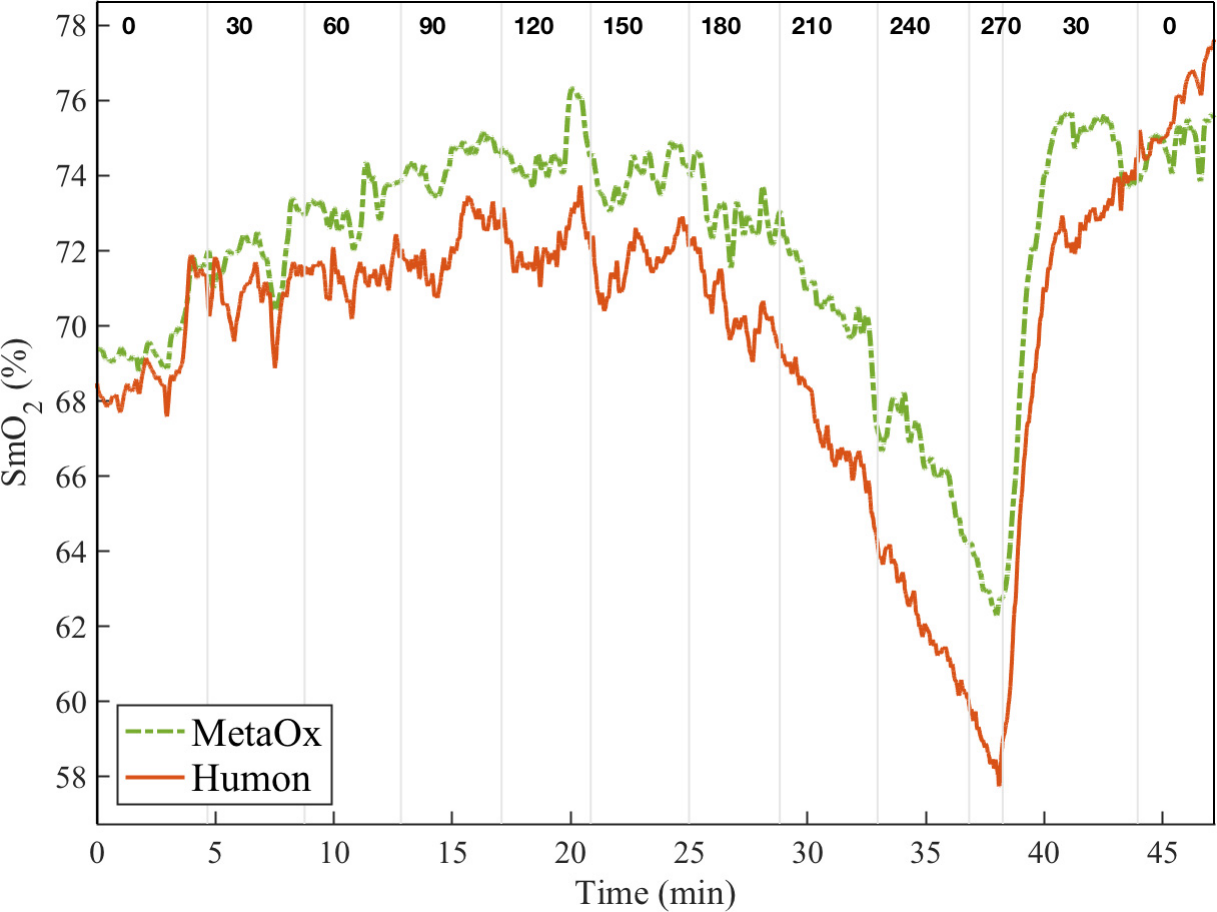
SmO_2_ results for a representative subject (subject #4) during the incremental cycling test. Humon Beta SmO_2_ (solid line) and MetaOx SmO_2_ (dashed line) absolute values are 3–5% different but the lines closely follow each other for the whole duration of the exercise. The vertical lines indicate the different cycling power periods with the power level indicated on the top.

We tested the accuracy of the HbO, HbR, HbT, and SmO_2_ estimations from the Humon Beta data against the MetaOx measurements. The data from all 17 subjects are included for these calculations. A representative dataset from the incremental power test is shown in Figure 3, where the change of SmO_2_ over time is presented for both MetaOx (dotted curve) and Humon Beta (solid curve). The vertical lines indicate the time point that the power on the bike was changed and the numbers at the top of the plot between the lines show the power (Watts) at which the subject was cycling. In general, at the beginning of the exercise, the muscle oxygen saturation slightly increases. As the power increases and the subject undergoes high exertion levels, the oxygen saturation starts to decrease. Finally, SmO_2_ sharply increases when the subject starts the recovery phase.

**Figure 4:**
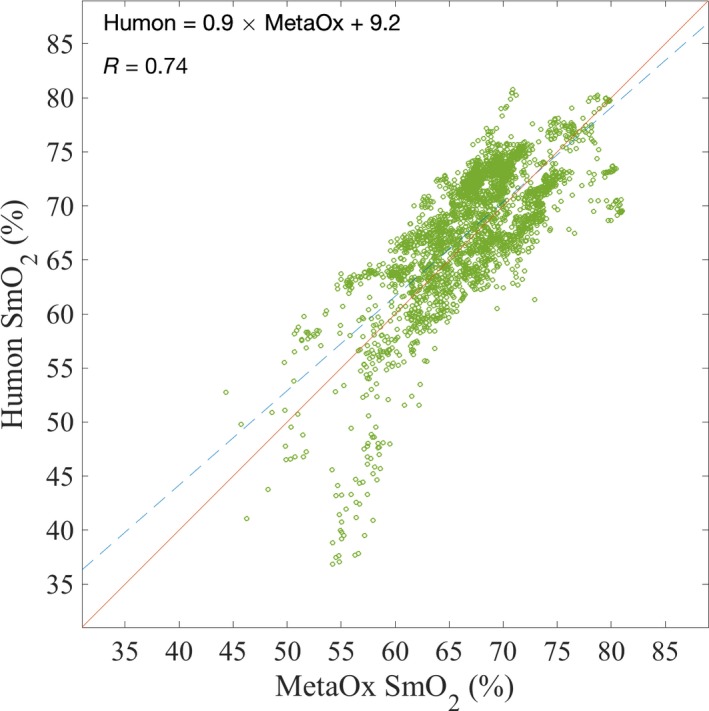
: Scatter plot of the SmO_2_ values measured by Humon Beta (*y*‐axis) and MetaOx (*x*‐axis) during exercise for all subjects. Data were down sampled to 12 sec per point, to simplify the figure. A strong linear relationship can be observed between the SmO_2_ measured by both systems. The equation and correlation coefficient of the best linear fit (dashed line) are reported in the figure.

Figure 4 is a scatter plot of the SmO_2_ values measured by the two devices in all subjects during cycling. The solid black line in Figure 4 indicates a line with slope of one and zero intercept, which represents the trend if there was a 1:1 relationship between the measured SmO_2_ by Humon Beta and the MetaOx. The dashed line shows the best linear fit to the data, with an intercept of ~10 and *R* = 0.74. This linear relationship was found to be significant (*P* value < 0.001 for slope and intercept). With respect to the MetaOx, the Humon Beta overestimates (2–3%) SmO_2_ at low values and underestimates (1%) SmO_2_ at high values.

### Quantifying differences between Humon Beta and MetaOx measurements

The results of the comparison between Humon Beta and MetaOx, MetaOx‐subset and MetaOx, and Humon Beta at two locations are reported in Table 2. In all three cases, the correlation coefficient and the relative RMSE between two measures are reported for HbO, HbR, HbT, and SmO_2_, averaged over all subjects.

**Table 2:** Results comparisons

Comparison	Parameter	Correlation coefficient	Relative RMSE (%)
Humon vs. MetaOx	HbO	0.80 ± 0.21	19.4 ± 14.4
	HbR	082 ± 0.19	25.7 ± 17.6
	HbT	0.72 ± 0.33	20.7 ± 15.6
	SmO_2_	0.86 ± 0.10	3.4 ± 0.9
MetaOx‐subset vs. MetaOx	HbO	0.89 ± 0.10	14.7 ± 10.9
	HbR	0.96 ± 0.08	14.7 ± 9.9
	HbT	0.83 ± 0.16	12.8 ± 8.7
	SmO_2_	0.98 ± 0.02	3.6 ± 1.9
Humon loc.1 vs.Humon loc. 2	HbO	0.86 ± 0.15	22.4 ± 15.1
	HbR	0.85 ± 0.16	16.4 ± 10.8
	HbT	0.92 ± 0.03	20.4 ± 14.6
	SmO_2_	0.87 ± 0.14	3.6 ± 3.0

The average of correlation coefficients and relative root mean square error (RMSE) of HbO, HbR, HbT, and SmO_2_ over all subjects comparing two different datasets as described in the first column. The comparison of Humon Beta at two locations on the muscle is performed on seven subjects, which had two Humon devices on the same muscle.

The results show a good agreement between Humon Beta and MetaOx data. The differences are larger for HbO, HbR, and HbT (19.4%, 25.7%, and 20.7%, respectively) and smaller for SmO_2_ (3.4%). While the absolute hemoglobin concentration values are different, a strong correlation (correlation coefficient: 0.72‐0.86) is present between the time traces measured with the Humon Beta and the MetaOx.

The MetaOx‐subset assumes the same fixed scattering coefficient and similar wavelengths and separations as the Humon Beta. Table 2 shows that the errors due to the simplified model results in a small deviation from the full MetaOx data. The average relative RMSE between MetaOx‐subset and MetaOx are 14.7%, and 14.7%, 12.8%, for HbO, HbR, and HbT, respectively, and 3.6% for SmO_2_ (correlation coefficient: 0.83–0.96).

To estimate differences due to differences in probe location on the same rectus femoris, the data from the two Humon Betas worn on the same leg (7 subjects) are compared and the results are shown in Table 2, Humon location 1 vs. Humon location 2.

Features within the SmO_2_ curves were also compared between the MetaOx and Humon Beta measurements for the 14 male subjects. Table 3 reports the average SmO_2_% drop throughout the cycling tests, the average time at which 50% of the recovery occurred, and the average SmO_2_% overshoot relative to the average starting value during the first power increment (30 W). A Wilcoxon rank sum test was performed between the MetaOx and Humon Beta data and the *p* values shown in the final row of Table 3 indicate that these values are not significantly different between the two systems (*P* values > 0.05).

**Table 3:** SmO_2_ feature comparison

	SmO_2_ Drop (%)	Time to 50% Recovery (sec)	SmO_2_ Overshoot (%)
*Humon*	11.9 ± 5.9	123.4 ± 39.7	8.2 ± 4.9
*MetaOx*	11.6 ± 5.8	100.8 ± 42.4	6.9 ± 3.7
*P value*	0.87	0.18	0.60

The SmO_2_% drop throughout the incremental test (first column); The average time to reach 50% of the recovery (middle column), and the average SmO_2_% overshoot (last column) are reported for the two systems. The p‐values indicate there are not significant differences between the measured parameters with the two devices.

### Determining the lactate threshold power

The SmO_2_ data for the 14 male subjects were analyzed to determine if the lactate threshold power could be found using MetaOx and Humon Beta SmO_2_ changes. Neither an SmO_2_ absolute threshold value or relative threshold drop could identify the lactate threshold power accurately. In particular, due to the large variability in SmO_2_ values across subjects, we could not find an absolute threshold value that could work for all subjects. Also, the difference between maximum SmO_2_ and SmO_2_ at 4 mmol/L lactate concentration (SmO_2_‐_drop_) varied a lot across subjects. At best, with either the MetaOx or the Humon Beta, by setting the threshold drop equal to the average SmO_2_‐_drop_ across subjects, we obtained an average power difference with respect to the blood lactate measure of about 40 W and a delay of about 5 min.

**Figure 5:**
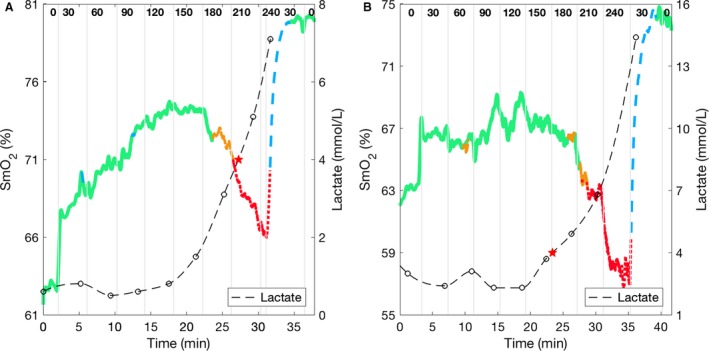
Two representative cases of Humon SmO_2_ with the estimated zones for (A) subject #2 and (B) subject #1. The measured blood lactate concentration is plotted in the right *y*‐axis (empty circles). The estimate 4 mmol/L blood lactate threshold is indicated by a red star. The vertical lines indicate the different cycling power periods with the power level indicated on the top.

Instead, the Humon Beta proprietary algorithm used to determine the lactate threshold showed good agreements with the blood lactate measurements. Representative cases of the Humon Beta SmO_2_ with the four exercise zones are shown for subjects one and two in Figure 5. The beginning of the red zone should correspond to the 4 mmol/L lactate concentration threshold. While the Humon Beta algorithms work well for subject #2, there is a relatively large delay on the estimate of lactate power in subject #1. We evaluated the accuracy of this threshold with respect to the blood lactate concentration measures in all 14 male subjects.

Table 4 shows the difference between the threshold power found by the blood lactate measurement (interpolating the data acquired every 4 min) and the threshold power determined by the Humon Beta proprietary algorithm in the 14 male subjects. The time difference between the two thresholds is also reported. Since the power increments during the cycling test are 30 W, the Humon Beta identified 4 threshold powers correctly and was off by 1 power increment (30 W) in 10 subjects. The absolute average power difference is 21.4 W and the absolute average time difference is 2:32 min.

**Table 4:** Comparison of lactate threshold power estimated by blood lactate concentration and Humon Beta proprietary algorithm

Subject #	Power Difference (W)	Time Difference (min:sec)
1	30	4:35
2	0	−0:44
4	30	1:06
5	0	−1:14
7	0	−1:25
8	30	3:06
9	30	1:55
10	30	4:37
11	0	−1:25
12	30	1:58
14	−30	−2:56
15	−30	−2:34
17	30	4:47
18	30	3:11
Absolute average difference	21.4 ± 14.1	2:32 ± 1:23

Difference between the threshold power found by the blood lactate concentration measurements and by the Humon Beta algorithm for the 14 male subjects, and the time difference between the two. The positive values indicate power and time delays of the Humon Beta with respect to the blood lactate. The last row reports the absolute average values.

### Influence of superficial layers on the measured SmO_2_

**Figure 6:**
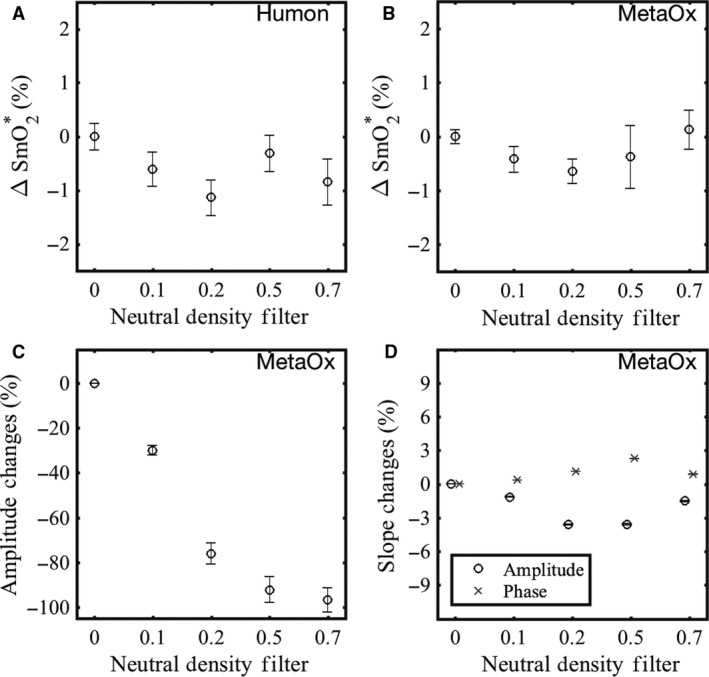
Effect of a superficial thin absorbing layer on measured optical parameters. Four different neutral density filters were positioned between the optical probe and a silicone phantom to mimic a thin attenuating superficial layer. Panels (A) and (B) report the difference between the effective hemoglobin saturation (SmO_2_*, the SmO_2_ we would have estimated if there was blood in the phantom) measured by Humon Beta and MetaOx with or without filter. The error bars represent the difference over five repetitions. Panel (C) shows the strong attenuation of the detected light at 3.0 cm source–detector distance at a representative wavelength (690 nm) in the MetaOx system. Panel (D) shows the corresponding amplitude and phase slope changes with the neutral density filters. Results are consistent at all wavelengths, independent of the absorbance spectra of the ND filters. In fact, ND filters have constant absorption in the visible spectral range, but they attenuate less in the near‐infrared range. This difference in attenuation spectra does not affect the effective SmO_2_ calculated using both red and near‐infrared wavelengths, since at each wavelength the effect of the ND on the slopes is negligible.

Using a silicone tissue mimicking phantom with known optical properties, we verified that neutral density (ND) filters minimally affect the recovered effective SmO_2_ as measured by the Humon Beta and MetaOx (Fig. 6 plots A and B). By placing neutral density filters of 0.1, 0.2, 0.5, and 0.7 optical densities over the phantom, the FDNIRS light intensity measured by the MetaOx is attenuated near 100% (Fig. 6C). In the linear fitting of intensity, amplitude, and phase versus distance, the thin absorbing layer affects the intercepts while the corresponding slopes are minimally affected (Fig. 6D). As a result, the recovered absorption and reduced scattering coefficients of the phantom are not impacted by the presence of the thin superficial absorber. Therefore, the recovered effective oxygen saturation (SmO_2_*) is not affected as shown in Figure 6B, where the SmO_2_* changes with respect to no filter are less than 2%. Similarly, Figure 6A shows that the SmO_2_ estimated by the Humon Beta, which also rely on multidistance algorithms, is not affected by the presence of the ND filters.

**Figure 7:**
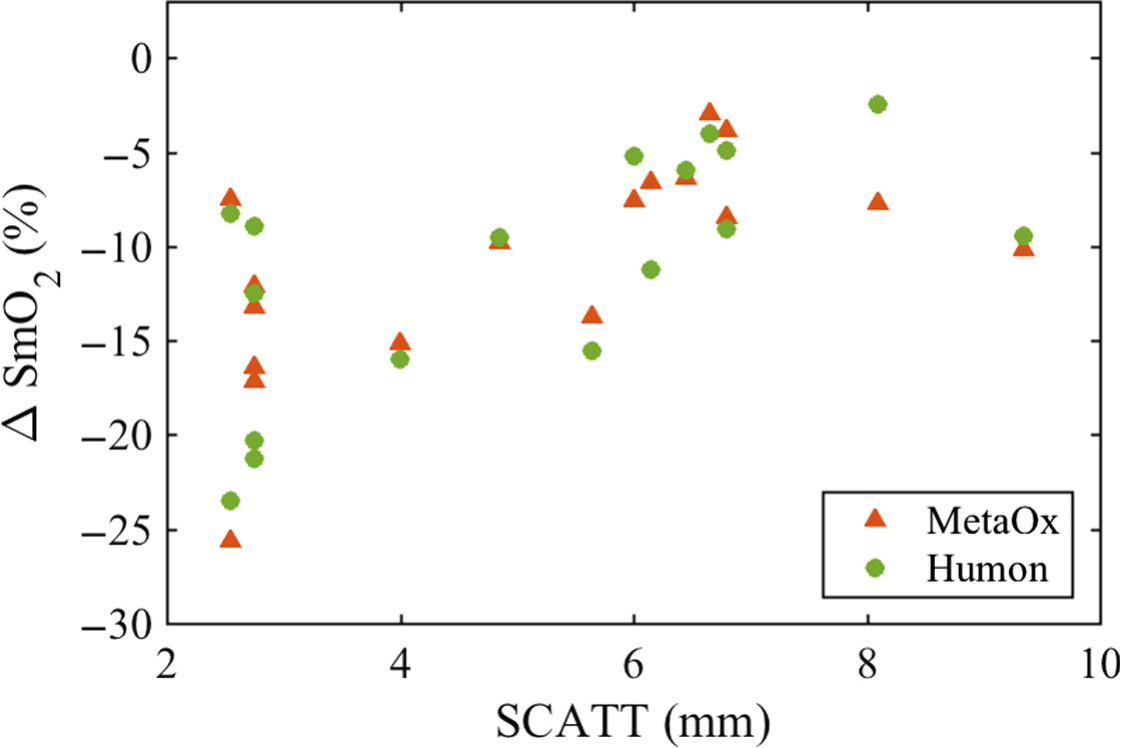
Scatterplot of SmO_2_ drop during exercise versus subcutaneous adipose tissue. With both devices, for thicker adipose tissue we consistently measured smaller drops in SmO_2_.

Finally, to evaluate the effect of subcutaneous adipose tissue on the estimated muscle hemoglobin parameters, we compared MetaOx results at shorter and larger separations. Using larger separations, we consistently obtained higher hemoglobin concentration. Across all subjects, the difference in total hemoglobin concentration is about 17%. Instead, the difference in SmO_2_ at the two separations is small (2.8% ± 1.4%). In addition, we found the SmO_2_ drop at the end of the exercise to be on average 11.8 ± 5.2% and 12.7 ± 5.5%, using the shorter and larger separations, respectively. By performing a Wilcoxon Rank Sum test, these drops were not found to be significantly different (*P* > 0.05). This indicates that the depth penetration is similar between the shorter and longer separations used. To further assess the effect of the subcutaneous adipose tissue on SmO_2_, we estimated the correlation between the total SmO_2_ drop (using all source–detector separations available in each system) and the SCATT in all subjects. As shown in Figure 7, we consistently measured smaller SmO_2_ drops for thicker subcutaneous adipose layers (with a correlation coefficient of 0.65).

## Discussion

In this study, we recorded NIRS data from 17 athletes while they were performing an incremental cycling test. The optically measured hemoglobin parameters (HbO, HbR, HbT, and SmO_2_) responded to muscle physiological changes during exercise. These results exhibit meaningful trends that can provide insights to how muscles respond to exertion. The average optical (absorption and reduced scattering coefficients) properties are illustrated in Figure 2. The measured values are consistent with values reported in the literature using similar NIRS methods to measure skeletal muscles (Yu et al. 2005; Gurley et al. 2012; Shang et al. 2012; Mesquita et al. 2013; Baker et al. 2017).

### Comparison of Humon Beta and MetaOx estimates of SmO_2_

During exercise, the body supplies a higher level of blood flow to the working muscles to provide oxygen (Yu et al. 2005; Joyner and Casey 2015). In the initial phase of cycling, when the athletes were working at a lower power, there was an overcompensation of blood, thus SmO_2_ slightly increased (Fig. 3 and Fig. 5). As the subjects approached their lactate threshold power, the SmO_2_ decreased, indicating that the oxygen consumption in the muscle had exceeded the oxygen supply. When the subjects could no longer continue pedaling at high power, and began the recovery phase, the SmO_2_ significantly increased due to the high blood supply and sudden decrease of muscle oxygen consumption. The agreement between the estimated SmO_2_ between the Humon and MetaOx system is consistent across all phases of exercise and all subjects as shown in Table 2 by the high correlation coefficient (0.86). Not only the trends, but also the absolute values on SmO_2_ are quite similar between the two systems, as shown by the low average RMSE (3.4%). Figure 4 shows that the range of variations of SmO_2_ measured with the Humon Beta system is smaller than the range measured with the MetaOx. Using the MetaOx subset model, we verified this difference is not due to the fixed scattering assumption. In fact, the *R* ^2^ between the two MetaOx models is 0.87 and the range of variation is actually larger by fixing scattering than by calculating it. Throughout the duration of the exercise, the scattering coefficients also changed minimally (on average less than 10%). The difference between the Humon and MetaOx estimated SmO_2_ is probably due to the different consumption of the right and left leg muscles. Eighty‐eight percent of our subjects were right‐footed and the MetaOx probe was measuring the dominant leg, therefore this may cause some discrepancies between measurements. Bilateral differences have been found by Hesford et al. (2012), who examined asymmetry between the SmO_2_ of the left and right quadriceps in ice skaters, and found differences depending on the muscle side that was being exerted more. The data from Table 3, supports the similarities between the features of the SmO_2_ curves measured on the 14 male subjects by the MetaOx and Humon Beta. We investigated the relationship between recovery time and maximum blood lactate concentration, and similar to Chance et al. (1992), we found these variables are uncorrelated. Chance et al. (1992) did find, however, that if individuals worked at a fraction of their maximum power level, there was a correlation between the recovery time and blood lactate accumulation. We cannot verify this finding since in our protocol we only measured recovery to maximum power output.

### Comparison of Humon Beta and MetaOx estimates of hemoglobin concentration

While there was strong agreement between the SmO_2_ of the two devices, we found larger differences in the hemoglobin concentration values. This result is somehow expected since the assumption of fixed scattering heavily affects the hemoglobin concentrations absolute values (Fantini et al. 1999). Nevertheless, we observed that the model error (fixed scattering, no phase information, only two wavelengths and only three separations vs. full FDNIRS MetaOx data), is smaller than the effect of muscle heterogeneity. As reported in Table 2, the RMSE between Humon and MetaOx are similar to the RMSE found comparing two Humon locations and double the RMSE found comparing different MetaOx models. This suggests muscle heterogeneity plays a larger role than differences between CW and FDNIRS models. The hemoglobin concentration spatial distribution throughout the muscle has been previously reported (Hamaoka et al. 2011), and it needs to be considered, when assessing muscle physiology with NIRS. Importantly, we found that while hemoglobin content significantly differs across locations, its temporal changes during the incremental cycling exercise are uniform across measured parts of the working muscle. In fact, in all our comparisons, we found good correlation coefficients for HbO, HbR, and HbT.

### Determination of the threshold power

On average SmO_2_ values at exercise onset in the 14 male subjects were 67.8 ± 4.0% for the MetaOx and 65.6 ± 4.9% for the Humon Beta. SmO_2_ increased during the initial power increments and at the lactate threshold has decreased 6.2 ± 4.6% and 6.3 ± 3.7% from the maximum SmO_2_, measured by the MetaOx and Humon Beta, respectively. Because of large variability across subjects, we could not find an SmO_2_ threshold value that could work for all subjects to estimate lactate threshold power (the best attempt found an average power difference of 40 W and a delay of 5 minutes). This is in agreement with previous NIRS works that show how the use of more complex analysis techniques of SmO_2_ in addition to HbR, HbO, and HbT are needed to better assess lactate threshold power from NIRS (Belardinelli et al. 1995; Grassi et al. 1999; Wang et al. 2006; van der Zwaard et al. 2016). The drop in SmO_2_ was larger when considering the maximum SmO_2_ measured during the low power intervals as the reference value. SmO_2_ at rest, before starting pedaling, was on average 3.5% lower than during the first power interval (30 W). We believe that in addition to the increase of blood flow with exercise onset which increases SmO_2_, the muscle contraction and skin tightening during the pedaling play a role here, reducing the contribution of superficial adipose tissue to the measured parameters. For this reason, to avoid this possible measurement artifact, in all our analyses, we only considered differences while pedaling, not with respect to rest.

The Humon Beta uses a combination of HbO, HbR, HbT, and SmO_2_ changes to identify the lactate threshold. We tested the agreement between the Humon estimated threshold with the one identified by the lactate concentration measures and found a close agreement between the two. The Humon Beta's real‐time threshold power on average only differs 21.4 W and less than 3 min from the invasive measure of blood lactate (Table 4). On the seven subjects with two Humon Beta devices, we also verified the time at which the threshold power was found was on average less than one minute apart between the two locations. The threshold power algorithms are proprietary to Dynometrics Inc. and are being refined as more data is collected in controlled exercise settings like this one.

Finally, testing the effects that a light attenuating thin superficial layer has on the NIRS data is crucial to ensure dependable results across a wide range of individuals with different melanin concentration in the skin. It is important to note that ND filters have constant absorption in the visible spectral range, but they attenuate less in the near‐infrared range. This difference in attenuation spectra does not affect the effective SmO_2_ calculated using both red and near‐infrared wavelengths, since at each wavelength the effect of the ND on the slopes is negligible. Both the MetaOx and the Humon Beta recovered correct effective SmO_2_ values across all the absorbing filters used. The reason why the effect of the filter is negligible is that both the MetaOx and the Humon Beta recover the optical properties from the gradient over distance, not from the absolute intensity values. This ensures confidence that the Humon Beta can measure SmO_2_ in people of all skin colors (Franceschini et al. 1998).

### Limitations

It is important to address that when validating the Humon Beta against the MetaOx, the NIRS data was obtained on different legs of the subjects. While cycling typically engages both legs uniformly, more differences may arise in the data because the two systems were not measuring the exact same location.

For the lactate power analysis, we had to exclude the female subjects, since we realized this protocol is not well suited for females. The power increments were too large, which resulted in the lactate threshold power and end of the exercise to be too close in time. We suggest smaller power increments for each step to be used when measuring female subjects who have smaller quadriceps compared to males.

The subcutaneous adipose layer influences quantification of hemoglobin parameters, especially at shorter source–detector separations. We verified that using larger (2.5–3.0 cm) source–detector separations relative to the shorter (1.5–2.0 cm) we obtain an average of ~17% higher hemoglobin concentration over the exercise duration. This is expected because of the larger penetration depth at longer separations and because of the larger hemoglobin concentration of muscle with respect to adipose tissue. As a result, in the presence of a subcutaneous adipose tissue layer, we measure higher hemoglobin concentrations at larger separations. Since fat and muscle have similar oxygenation at rest, the difference in SmO_2_ at the two separations is small (2.8% ± 1.4%). We verified that during exercise, the difference in SmO_2_ at the two sets of distances is constant and did not have statistically significant differences in the SmO_2_ drops by the end of exercise. Nevertheless, at both sets of distances, the presence of a subcutaneous adipose tissue layer affects the measured SmO_2_ as shown by the correlation between the SmO_2_ drop and the SCATT in Figure 7. For thicker SCATT layers, there are smaller drops in SmO2 since the volume measured includes less muscle and more fat. For the specific application in athletes and fit individuals, the SCATT is low, reducing the contamination by the adipose tissue. While we only measured the SCATT over the rectus femoris of the right leg, we also assumed that the SCATT is symmetric and that this layer reported in Figure 7 affect similarly the MetaOx and Humon Beta data.

In this study, we chose to monitor the rectus femoris rather than the vastus lateralis muscle (which is the common choice in other work examining NIRS measurements in sports (Perrey and Ferrari 2018) to minimize optical fiber movement throughout the cycling exercise. Further work needs to be done to examine the differences in SmO_2_ measured in multiple muscle groups simultaneously, using several Humon Beta devices to help understand what muscle group is the optimal choice for certain types of activities. It is also crucial to determine if the identification of an athletes’ threshold by NIRS is dependent on the exact muscle chosen from the many working muscles during a sport.

When comparing the NIRS measurements against the lactate threshold, it is relevant to mention that multiple methods exist to find the lactate threshold (Faude et al. 2009). By choosing a different threshold identification technique (besides 4 mmol/L), a new threshold power may be identified, which may alter the values in Table 4. We chose to determine the lactate threshold as 4 mmol/L because of the widespread use and established studies that support this method (Heck et al. 1985; Faude et al. 2009).

## Conclusion

In summary, this study validates the performance of a low‐cost, wireless, wearable NIRS device against an advanced benchtop device. During the incremental cycling test, the wearable device provides similar results to the more expensive FDNIRS technology. We verified that the assumptions and simplifications of this CWNIRS system minimally impact the SmO_2_ quantification and the recovery of changes in the hemoglobin concentration trends. The main deviations are accounted for by muscle heterogeneities. While skin color does not affect the results, the main limitation, common to all CW and FDNIRS systems, is the reduced sensitivity to muscles in the presence of subcutaneous adipose tissue. Targeting athletes, who tend to be fit individuals with thin adipose layers, provides a larger drop in SmO_2_ readings than what can be achieved on people with thicker adipose thicker layers. Finally, we demonstrated the Humon Beta shows a good accuracy in predicting the lactate threshold level. Thanks to the low cost, small size, and low sensitivity to motion, the Humon Beta wearable and its accompanying smartphone application can provide useful information about exercising muscle physiology and help athletes optimize their training.

## Conflict of Interest

Dr. Franceschini has patents on the FDNIRS technology used in this paper.
